# Cognitive dysfunction—an under looked avenue to promote health in incarcerated elderly population through yoga

**DOI:** 10.3389/fnhum.2025.1553845

**Published:** 2025-05-09

**Authors:** Kalyan Maity, Vijaya Majumdar, Sanjib Patra, Akshay Anand

**Affiliations:** ^1^Division of Yoga and Life Sciences, Swami Vivekananda Yoga Anusandhana Samsthana (S-VYASA), Bengaluru, India; ^2^Neuroscience Research Lab, Department of Neurology, Post Graduate Institute of Medical Education & Research, Chandigarh, India; ^3^Department of Yoga, Central University of Rajasthan, Ajmer, India; ^4^CCRYN - Collaborative Centre for Mind Body Interventions Through Yoga, Post Graduate Institute of Medical Education & Research, Chandigarh, India; ^5^Centre of Phenomenology and Cognitive Sciences, Panjab University, Chandigarh, India

**Keywords:** cognitive impairment, yoga, prison, elderly, ageing crisis

## Abstract

The correctional “ageing crisis” underlined by accelerated ageing, attributed to a prior history of poor health and lifestyle choices amongst prisoners, and other imprisonment-related factors has been associated with a global burgeoning burden of cognitive impairment in prison settings. Cognitive impairment imposes a crucial, urgent economic and medical challenge for carceral healthcare systems. Further, lack of awareness and absence of regular diagnostic screenings and lack of implementation of appropriate interventions in the prison settings worsen the scenario. Amongst the limited efficacy and reported side effects of pharmacological treatments for cognitive dysfunction, Yoga, a cost-effective and scalable intervention could provide better avenues for halting cognitive deterioration in elderly prisoners. This article presents a perspective on how the adaptation of Yoga-based regimes in carceral settings could improve the needs of people with cognitive deficits across Indian prison settings. However, we also emphasize the need to understand the essence of readiness to achieve clinical effectiveness for combating cognitive deterioration, via the implementation of well-structured Yoga based rehabilitative programs.

## 1 Introduction

Poor health profile is a general characteristic feature of prisoners across the globe (Fazel and Baillargeon, 2011). The increased burden of wide spectrum of diseases identified in incarcerated population proves the need of holistic care models instead of only treating to the disease in order to manage extensive comorbidities and to support the alongside treatment (Edge and Hayward, 2024). Amongst a spectrum of health issues, correctional settings impose a much higher load of psychiatric morbidities among prison inmates compared to the general population, with a pooled estimate of reattributed to the bi-directionality of cause and effect between mental health illnesses and crime (Ayirolimeethal et al., 2014; Dean et al., 2018; Yukhnenko et al., 2023). Studies demonstrated around 35% of under-trial prisoners have prior psychiatric conditions before offending (Mohan and Dhar, 2001).

Crime is, in general, a manifestation of risk traits for compromised cognitive functioning including poor cognitive control, paranoid ideation, poor judgmental ability, hallucinations and delusions (Bantjes et al., 2020; Dhillon and Sasidharan, 2024). Further, amongst cognitive dysfunctions, executive function, is a documented risk factor for criminal behavior (Morgan and Lilienfeld, 2000; Ogilvie et al., 2011). However, Cognitive dysfunction usually remains undiagnosed and untreated in correctional settings.

Prisons settings provide an important opportunity to address health inequalities by providing services to underserved high-risk populations (Edge and Hayward, 2024). Hence, if tapped effectively, incarceration could be utilized as a unique time interval to diagnose, assess and treat the heterogeneous health needs of this underserved populations (Favril et al., 2024).

### 1.1 Global trends of research on incarcerated elderly prisoners and associated cognitive deficits

The recent trends of an alarming rise in the prison population by 23% from 2019 to 2021 draw attention to the concurrent global rise in the number of incarcerated elderly prisoners, in particular with the alarmingly high raise of approximately 400% since 1993 (Carson and Sabol, 2016). The increase number of elderly prisoners in different countries has been described as a correctional “ageing crisis” which poses an urgent economic and medical challenge for carceral health-care systems (Wangmo et al., 2016). The ageing crisis also carries a burden of overt manifestation of ageing-related cognitive dysfunctions, majorly associated with executive dysfunction, memory impairments, and attention deficits (Meijers et al., 2015; Rocha et al., 2015).

As of now, most of the research on mental health problems among prisoners has been conducted in high-income countries (HICs). Prisoners with the age of ≥55 years have around 35% mild cognitive impairment (MCI) and 9.1% dementia in the United States (Baillargeon et al., 2023). Whereas the general US population with age between 65 and 74 years showed 5.3% of Alzheimer’s disease (AD) and 21.9% of MCI, age between 75 and 84 years showed 13.8% of AD and 24.6% of MCI, and age over 85 years showed 34.6% of AD and 22.1% of MCI (Rajan et al., 2021). This indicates that the general population can develop cognitive deficits above 85 years of age and that could be developed at the age of around 55 years in prisoners. Around 15.56% of the general population aged ≥50 years have MCI globally (Bai et al., 2022), which is lower than the prisoners in a similar age group, and indicates a higher risk of MCI in the prisoners compared to the general population. Dementia or related cognitive impairments do not manifest in isolation, but together with several behavioral symptoms, namely physical aggression, impulsiveness, and mood lability (Lyketsos et al., 2002; Peters et al., 2006). This may possibly lead to elderly individuals experiencing arrest and imprisonment due to property theft or encroachment, and traffic violations (Liljegren et al., 2015; Talaslahti et al., 2021). Cognitive impairment could be accelerated by several catalysts, in particular, psychological factors such as stress, anxiety, and depression leading to the onset of early cognitive deficits (Avdija, 2022). A 12-year follow-up study has shown that chronic stress can convert general people into MCI and a higher level of stress is associated with a higher percentage of MCI (Wilson et al., 2007). On the other hand, the conversion rate from MCI to AD or dementia is around 23.8%, and one person among four is at risk in low and middle-income countries (McGrattan et al., 2022). Interestingly, recent evidence has shown a direct causative association between dementia and stress, wherein authors reported 2.20 times higher rate of dementia in stress diagnosed individual compared to no stress diagnosed individuals (Islamoska et al., 2021). Also, anxiety and depression are considered a predictor of cognitive impairment and can be considered a risk factor for cognitive deficits (Freire et al., 2017). A very recent study on around 40,000 UK Biobank participants have shown significant genome-wide associations between brain network and seven genetic clusters linked with cardiovascular deaths, schizophrenia, Alzheimer’s and Parkinson’s disease (Manuello et al., 2024). The most vulnerable modifiable risk factors for this are diabetes, nitrogen dioxide due to air pollution, alcohol consumption (Manuello et al., 2024). The Lancet commission has updated the 12 modifiable risk factors for dementia that includes diabetes, hypertension, head injury, smoking, air pollution, midlife obesity, lack of exercise, depression, alcohol consumption, hearing impairment, social isolation, lack of education which may lead to 40% of dementia globally and reduction of these may prevent dementia (Livingston et al., 2020). Cognitive impairment and dementia are associated with criminal behavior (Morgan and Lilienfeld, 2000; Wallinius et al., 2019). Indirectly these risk factors may responsible for criminal behavior and imprisonment.

As observed in general trends in research, there remains a scarcity of research data from low and middle-income countries (LMICs) like India. Notably, these countries exhibit a mental health gap, defined as the difference between the number of individuals with mental illnesses and those who have access to appropriate treatment – with estimates as high as 85% compared to only 40% in HICs (World Health Organization, 2016). Such alarming figures of mental health gap represent worrisome health care trends in particular, for the vulnerable population such as prisoners, with already meagre access to mental health resources and substantial delays in referral and treatment gaps in mental health evaluation (Torrey et al., 2014).

### 1.2 The Indian scenario

Research on prison mental health is relatively rare in India than in other countries, with a reported prevalence of mental health problems ranging from 21 to 33% among prisoners (Dhillon and Sasidharan, 2024). The disparity in prevalence rates has been attributed to the use of different assessment tools used for estimation of prevalence rates of different disorders. Despite being the second-most populous country globally, only 9,000 psychiatrists are present as per the National Mental Health Survey of India, 2016, translating up to 0.75 psychiatrists/100,000 population, the required number being only above 3 psychiatrists per 100,000 (Garg et al., 2019) resulting in a vast mental health gap ranging from 75 to 93% in the general population (Demyttenaere et al., 2004; Patel et al., 2018; Sagar et al., 2017).

The latest report of the Prison Statistics India-2022 projects an alarming scenario with approximately 25.6% of prisoners being illiterate, majority being homeless, shunned by their families/communities, and with history of sustained physical/sexual violence/exploitation (Prison Statistics India, 2022). Compounding the same, the atmosphere inside Indian prisons is repugnant due to overpopulation, dearth of privacy, lack of facilities, skepticism about the future, and extensive torturous practices predisposing various mental and physical disorders such as tuberculosis and HIV infections (Dolan and Larney, 2010; Prasad et al., 2017). Overall, the cumulative effect of existing treatment gap, the prison statistical estimates showcasing raise in elderly and their poor health profile and the prison environment impose severe risk of aging crises and associated cognitive dysfunction in the Indian prisons. Adding to the same cognitive evaluation in the Indian prisons remains limited by an array of ethical and logistical challenges in terms of conducting cognitive assessments (Aggarwal and Joseph, 2020).

Realizing the same the Ministry of Home affairs, the Government of India is running several schemes for education oriented rehabilitative and vocational training for Indian prisoners. A total of 1,04,623 prisoners had been educated in the country during the year 2022. Among them 46,786 prisoners were received elementary education, 39,888 prisoners were received adult education, 12,780 prisoners were received higher education and 5,169 prisoners were educated in the field of Computers. Vocational Training includes weaving, tailoring, carpentry, agriculture, making soap and phenyl, handloom, canning etc. (Prison Statistics India, 2022). India already had the “Prisons Act, 1894” which mentions about the “Prisoners Right to health” ensuring the health of the prisoners. The central government of India has also updated this Act and brought out the “Model Prison Act, 2023” to facilitate the health care of the Indian prisoners focusing on their transformation, rehabilitation and reintegration into the society. The “All India Model Prison Manual Committee” further developed a “Model Prison Manual” for the effective management of prisoners (Devi, 2024). Recommendation were made towards effective implementation of the manuals in all the states and union territories, and some of the state governments have taken the initiatives to update their “Prison Manual” (Devi, 2024). Several other activities to boost healcare and sanitation of prisoners have also been parallelly implemented such as the *Swachh Bharat Abhiyan* and the Yoga, and meditation program for the inmates (Prison Statistics India, 2022). We also find successful celebrating of International Day of Yoga (IDY) across different prisons of India. These initiatives could be strengthened by implementation of disease-specific Yoga protocols across mental and physical health domains with particular emphasis on cognitive health of the elderly. Though pharmacological interventions play a crucial role in managing cognitive dysfunction, the existing treatment options remain limited by reported modest benefits, inability to reverse cognitive decline, and reported side effects like nausea, vomiting, and dizziness, limiting the tolerability and adherence of the drugs (Husain et al., 2008). For example, a recent study described the fall-related risks of medications generally used for cognitive enhancement or treating neurodegenerative diseases (Portlock et al., 2023). A very recent study on mice models has shown that Glutamine supplementation can prevent stress-induced MCI (Baek et al., 2020) but Glutamine supplementation on the pre-converted MCI population is unclear. Also, human studies are required to draw a clear conclusion. Given the dearth of pharmacologic or dietary agents to have symptomatic cognitive benefit in treating MCI and the lack of FDA (Food and Drug Administration) approved medications (Petersen et al., 2018).

Cognitive Training (CT) may be effective for cognitive enhancement in older prisoners, but the small sample size, less follow up duration and frail study design has given a chance to study the effectiveness of CT in older prisoners and explore further (Verhülsdonk et al., 2023a; Verhülsdonk et al., 2023b). Cognitive behavioral therapy (CBT) could be an another effective treatment for prisoners (Saxena and Sahai, 2024), and may reduce recidivism risk around 20 to 30% (Beaudry et al., 2021). However, studies related to CT and CBT in Indian prisons are rare. Over the years, advanced lifestyle-based combinations of diet, exercise, and mindfulness-based interventions have emerged as important treatment modalities against cognitive dysfunction (Capurso et al., 2019; Hastings et al., 2024; Snigdha et al., 2024). Yoga, a cost-effective and scalable intervention could provide better avenues for halting cognitive deterioration in elderly prisoners. However, there remains scarcity in literature on Yoga being introduced in the Indian prison setting, in context of promotion of cognitive health and halting the progression of cognitive impairment in the sector of vulnerable population (Arya et al., 2022; Baskaran, 2015; Nanduri and Ram, 2020; Sureka et al., 2014). The existing literature also indicates lack of rigor in study design including small sample size and shorter study durations (Sathiyavathi et al., 2024), further leading insignificant study outcomes (Arya et al., 2022).

## 2 Materials and methods

In this perspective, we have evaluated the influence of Yoga on cognitive function especially in prisoners. We have used search engines, PubMed and Google Scholar to search the literature by using combination of different keywords thread such as (“Yoga” or “Meditation” or “Mindfulness” or “Yog” or “Pranayama” or “Breathing practices”), (“Prison” or “incarceration” or “Jail” or “Imprisonment”), (“Cognitive impairment” or “Dementia” or “Alzheimer”) and (“MRI” or “fNIRS” or “Brain imaging”) without using any filters. Studies including clinical trials, randomized control trials, reviews, systematic reviews, and meta-analysis were included in this study.

## 3 Discussion

### 3.1 Biopsychosocial framework of yoga for cognitive dysfunction

In this article we present a connotation on Yoga for alleviation of cognitive declined associated with aging supportive by a proposed biopsychosocial framework built with biological (neurocognitive structural and functional influences), and psychosocial components (emotion regulation, stress reduction, self-control, recidivism, mindfulness, and social connectedness).

#### 3.1.1 Neurocognitive structural and functional influences

We find moderate-level of significant effect of Yoga on brain structure and neurocognitive functions in a wide spectrum of population ranging from healthy individuals to cognitively compromised elderly (Hariprasad et al., 2013; Santaella et al., 2019). Several studies have shown greater gray matter volume in specific brain regions like frontal, temporal, and occipital lobes, hippocampus, and cerebellum among Hatha Yoga meditation practitioners with more than 3 years of practicing experience compared to the naive control, indicating the better executive and cognitive functions.

At anatomical level, the practice of Yoga appears to be linked to frontal cortex, anterior cingulate cortex, hippocampus and insula, with positive relationship with different measures of brain structure such as gray matter volume, density, and cortical thickness (Gothe et al., 2019). Hippocampal volume increase is one of the majorly reported anatomical change following Yoga practice, linking its effects to learning and memory processes (Squire, 1992). Also, Yoga practitioners have shown significantly lesser chances of cognitive failures (Gothe et al., 2019). Recent studies have shown that Yoga may prevent gray matter atrophy (Krause-Sorio et al., 2022) and have neuroprotective effects on healthy elderly women practicing meditation at least for 8 years has shown greater cortical thickness in the left prefrontal regions (Afonso et al., 2017). A study on healthy older adults increased right hippocampal volume following 12 weeks of Kundalini Yoga practice indicating the neuroprotective and neurocognitive role of Yoga practice (Ibrahim et al., 2022). Even though the gray matter volume in the hippocampus region is greater among Yoga practitioners with more than three years of Yoga practice experience however, the potency of Yoga to maintain brain functions associated with executive function and working memory is still under development and has shown less activation of the dorsolateral prefrontal cortex compared to the control measured through functional MRI (Gothe et al., 2018). When reviewed for functional connectivity of the brain, authors reported Yoga practice to be positively associated with increases in the default mode network, and changes related to memory performance (Gothe et al., 2019). Another study has shown that 12 weeks of Yoga may improve stress-related hippocampal connectivity in women at risk for AD (Kilpatrick et al., 2023). A study by Garner et al. have shown a significant increase in right hippocampal grey matter density following 10 weeks of hatha Yoga intervention (Garner et al., 2019). A functional near infrared spectroscopy (fNIRS) based study has shown increased oxy-hemoglobin and reduced deoxy-hemoglobin during meditation practice in the right prefrontal cortex. Also, a shorter reaction time measured through the Stroop color-word task and a reduction in total hemoglobin concentration was observed after the meditation indicating better cognitive performance (Deepeshwar et al., 2015). On the other hand, 8 years of Yoga practice has shown greater functional connectivity between the medial prefrontal cortex (MPFC) and right angular gyrus (AGr) (Santaella et al., 2019).

At behavior level, acute and beneficial effects of Yoga on cognition supported by meta-analysis have been reported with moderate effect sizes for attention, processing speed and executive function measures for studies conducted with adult populations (Gothe et al., 2019).

Despite the established links between Yoga and brain health also supported by systematic review (Gothe et al., 2019), as stated earlier in this article, we find scarcity of data in India on effects of Yoga on cognitive health in prison settings, drawing the unmet need of its implementation in prisoners from the viewpoint of cognitive health (Sureka et al., 2014).

#### 3.1.2 Social connectedness

Social isolation standout as a major risk factor for the cognitive decline in prisoners. Prisoners stay in social isolation and feel lonely (Pageau et al., 2022) which might increase the risk of cognitive decline. A three-year follow-up study has shown that social isolation is significantly associated with lower cognitive functions tested through verbal fluency and forward digit span tests (Lara et al., 2019). A two-year follow-up study has shown that maintaining a socially active lifestyle may improve cognitive reserve in later life (Evans et al., 2018). On the other hand, a meta-analysis has shown that people with large social networks are associated with better cognitive functions in later life (Evans et al., 2019). An 11.7-year follow-up study on 462,619 participants has shown that social isolation may increase the risk of dementia by around 1.26-fold (Shen et al., 2022). Also, socially isolated people had lower gray matter volume in frontal, temporal, and hippocampal regions indicating under-expression of genes which is downregulated in AD (Shen et al., 2022).

Yoga may improve social interactions as evidenced by different studies and in improving the social domain of quality of life (Dhawan et al., 2024). Usually, Yoga interventions are administered in a group which may increase their social connectivity and interaction. A study has shown that 40 days of breathing-based Yoga intervention increases social connectedness, as measured by the social connectedness scale (Kanchibhotla et al., 2024). Integrated Yoga has shown to improve quality of life and interpersonal relationships (Rakhshani et al., 2010).

#### 3.1.3 Emotion regulation

Biologically social isolation has been known to release certain neuropeptides including BDNF that in turn leads to escalation of aggressive behavior (Takahashi, 2022). Elderly who has “aged in place” in prison owing to their long term incarceration (Bond et al., 2005). This is in contrast to the otherwise well documented positive affective experiences reported by elderly population. Hence, Yoga based intervention with a component of group-based format promoting social connectedness could aid in regulation of emotional turbulence experienced by prisoners also exhibiting aggression and antisocial behavior (Fazel et al., 2016; Seid et al., 2022). Our postulate is further supported by the reported effectiveness of Yoga in improving perceived stress, sleep quality, psychological and emotional well-being, aggressive and antisocial behavior of prisoners (Kerekes et al., 2017).

#### 3.1.4 Stress reduction

Incarceration, is in general associated with mental distress; prisoners exhibit varying degrees of psychiatric symptoms such as posttraumatic stress disorder (PTSD) ranging from 0.1 to 27% for males, and from 12 to 38% for female prisoners (Baranyi et al., 2018). Around 39.2% of prisoners have depression in developing and 33.1% of depression in developed countries (Bedaso et al., 2020). India is a developing country and has shown around 8% of stress, 8% of anxiety, and 18.5% of depression among prisoners which is much higher than the general population (Malik et al., 2019). Hence, prisoners are at higher risk for cognitive impairment mediated by stress, anxiety, and depression and need further exploration of cognition-based studies in prison settings. Yoga may reduce stress among prisoners (Kerekes et al., 2017) by reducing cortisol level, a well-established measure to study the HPA axis and positively correlates with psychological stress (James et al., 2023). However, most of the studies have investigated only male prisoners or female prisoners with a minimum sample size.

### 3.2 Considerations for implementation of yoga in prison settings

While research suggests Yoga offers promise for improving cognitive health in prisoners, successful implementation requires careful consideration of several factors: we hereby postulate a few implementation considerations for Yoga programs in prisons: optimizing for cognitive health (Table 1).

**Table 1:** Implementation considerations for yoga programs in prisons: optimizing for cognitive health.

Sr. No	Factors	Approach	Implementation related consideration
1.	Instructor training	Trauma-informed approach	Instructors should be trained in trauma-informed Yoga practices. Many prisoners have experienced trauma, and a sensitive approach that emphasizes safety and choice is crucial.
		Cognitive enhancement expertise	Instructors could benefit from additional training on Yoga practices specifically designed to target cognitive function. This may include memory-enhancing postures or meditation techniques focused on concentration.
		Cultural competency	Yoga instructors should be culturally competent to ensure the program is accessible and respectful of diverse backgrounds within the prison population.
2.	Program design	Focus on self-regulation	The program should emphasize practices that promote self-regulation skills, such as controlled breathing exercises and mindfulness meditation. These can help prisoners manage stress, improve focus, and enhance cognitive flexibility.
		Progression and differentiation	Design a program that allows for progression and caters to varying physical abilities and cognitive needs within the prison population. Offering beginner, intermediate, and advanced levels can encourage long-term engagement.
		Integration with cognitive rehabilitation	Explore potential integration with existing cognitive rehabilitation programs for a more holistic approach to cognitive health improvement.
3.	Sustainability and Support	Post-release support	Offer resources or programs that help prisoners maintain a Yoga practice after release. This can be crucial for sustained cognitive benefits and overall well-being.
		Peer support groups	Establish peer support groups within the program to foster a sense of community and motivate continued practice.
		Program evaluation	Implement a program evaluation plan to track the impact of Yoga on cognitive health in prisoners. This data can inform future program development and secure continued funding.

### 3.3 Additional considerations

#### 3.3.1 Space and equipment

Ensure adequate space and basic equipment (Yoga mats, blocks, straps) should be available for safe and effective Yoga practice.

#### 3.3.2 Scheduling and accessibility

Schedule classes at convenient times and consider offering modified Yoga practices for prisoners with limited mobility.

#### 3.3.3 Standardization of yoga protocol/ intervention

Interestingly, many Yoga studies in prison settings evaluated the effect of *Asana* (Posture), *Pranayama* (breath control), and *Dhyana* (meditation) but other elements of *Astanga* Yoga (8 elements of Yoga) are considerably used. *Astanga* Yoga includes *Yama* (self-restraints), *Niyama* (rules), *Asana* (Posture), *Pranayama* (breath control), *Pratyahara* (sense withdrawal), *Dharana* (concentration), *Dhyana* (meditation), and *Samadhi* (self-absorption). Hence, Yoga protocols need to be updated in holistic manner rather than only physical posture, they should include all other elements of *Astanga* Yoga. Also, Yoga studies with cognitive measurements in prison settings are rare (Bilderbeck et al., 2013). Hence Yoga protocol contains all the components of Yoga on cognitively impaired prisoners are need to be further explored. Before implementing this protocol, it should be validated by the various experts in the field and suggestions must be incorporated. Then this verified Yoga protocol can be implemented in the prison population.

#### 3.3.4 Intervention frequency

Most of the studies have shown less intervention frequency for example only a single Yoga session per week or twice per week and need further attention (Kerekes et al., 2017). At the same time, Yoga-based Indian prison studies are occasional (Arya et al., 2022). To the best of our knowledge, we did not find any molecular studies related to stress and cognition in prison settings.

#### 3.3.5 Classification based on age

Also, most of the prison-based studies included mixed age groups (Bilderbeck et al., 2013). As age can be a predictor for cognitive decline hence, further studies are required with age-wise classification.

#### 3.3.6 Classification based on traditional personality traits and sleep quality

Implementation of Yoga protocol for a particular person by understanding the nature of that person is very important. To do so larger studies can be planned by categorizing the participants based on their personality (*Sattva, Rajas, Tamas* and *Vata, Pitta, Kapha*) and preparing Yoga protocol accordingly. Also, poor sleep quality is a major contributor to cognition decline, hence prisoners can be categorized based on their sleep quality and design Yoga protocol accordingly.

#### 3.3.7 Precise study design

Prison based cognitive studies should adopt randomized controlled trials with probability or random sampling methods in order to ensure true representation of the population. Studies should used validated assessment tools such as mini-mental state examination (MMSE) (Arevalo-Rodriguez et al., 2021), montreal cognitive assessment (MoCA) (Nasreddine et al., 2005) for cognitive assessment. Each stage of research methods like data collection, Lab based experimentation, data analysis should be blinded. Researcher must ensure that there is no interaction between the participants of experimental and the control groups. Analysis procedure must be decided before commencement of the study. Good clinical practices or good lab practices should be followed (Anand, 2021).

#### 3.3.8 TIDieR and CLARIFY guidelines

Yoga research outcome should be reported based on TIDieR (Template for intervention description and replication) (Hoffmann et al., 2014) and CLARIFY (consensus-based CheckList stAndardising the Reporting of Interventions For Yoga) guidelines (Moonaz et al., 2021).

#### 3.3.9 Community-level implementation

Research on prisoners provides a sturdy platform for healthcare interventions due to minimum confounding by factors such as similar lifestyle (food, sleeping time, environment, etc). Hence, findings derived from research in prison settings could be directly extended to the prison community setting across the globe with considerations for culture-related differences. Further it may help in development of Yoga protocols catering to the specific needs of prison populations. Additionally, different community services (food preparation, making furniture for official setups, assisting in health education, health awareness camps, deaddiction centers) during imprisonment or post-release, are a few key socially relevant behavioral strategies that need rigorous implementation.

By addressing these implementation considerations, Yoga programs in prisons can be optimized to effectively promote cognitive health and well-being among incarcerated individuals. Additionally, the social aspects of group Yoga classes can combat feelings of isolation often experienced by prisoners, potentially reducing stress and fostering a sense of community, which can further benefit cognitive health.

### 3.4 Future directions and policy discussion

#### 3.4.1 Research in prison

Government should facilitate more integrative research across fields of mind–body medicine, psychiatry, and modern medicine in prison settings.

#### 3.4.2 Cognitive screening in prison

Diagnostic gap can be reduced by implementing frequent cognitive screening starting from the day of imprisonment followed by every three months for every prisoner using validated cognitive screening tools such as MMSE (Arevalo-Rodriguez et al., 2021), and MoCA (Nasreddine et al., 2005).

#### 3.4.3 Promoting the field of psychiatry and increasing the number of psychiatrists

There is an unmet need to nurture young medical professional to orient for the relevance of mental health care in India and to promote them adopt their mainstream medical field as psychiatry. This approach will facilitating mental health through increasing number of psychiatrists in the community or hospitals to indirectly help to reduce crime rate by getting mental health facility before crime. Consequently, there will also be an increased number of psychiatrists in the prison settings needed to fulfill mental health gap, facilitate cognitive screening and prevent cognitive impairment.

#### 3.4.4 Management of risk factors

Stress, anxiety and depression are major risk factor of cognitive impairment in prison. Implementation of different stress management program such as Yoga, mindfulness, and meditation could help to reduce cognitive dysfunction in prison (Lyons and Cantrell, 2016; Sureka et al., 2014) Government should reform “Prison Manual” with inclusion of mandatory Yoga program in prison.

#### 3.4.5 Medical curriculum in prison

Medical curriculum in prison should increase the awareness about the physical and mental health and hygiene. Moreover, it will help to prevent physical and mental morbidities. Infectious diseases are very common in Indian prison due to over-crowed and sanitation problems (Devi, 2024).

#### 3.4.6 Intervention at the transition phase

The transition phase is very crucial in which the prisoners are released from the prison and reintegrated in the community. However, research in this area is under developed (Hopkin et al., 2018). Hence, Government should developed specific interventions such as practice of Yoga protocol prior release of the prisoners to ensure their successful reintegration with the community to reduce recidivism (Kovalsky et al., 2021).

## 4 Conclusion

Though the scientific evidence on the effects of Yoga in cognitively impaired older adults endorses it as a safe and feasible treatment, there is a need for implementation of the same in prison populations with cognitive deficits. The well-structured Yoga protocols should be specific and customized based on the personality of prisoners. However, we also emphasize the need to understand the essence of readiness to achieve clinical effectiveness for combating cognitive deterioration, at national policy level with local strategies supporting a multi-disciplinary approach.

## Data Availability

The original contributions presented in the study are included in the article/supplementary material, further inquiries can be directed to the corresponding author.
