# Correction: Cognitive dysfunction—an under looked avenue to promote health in incarcerated elderly population through yoga

**DOI:** 10.3389/fnhum.2025.1725397

**Published:** 2025-10-27

**Authors:** Kalyan Maity, Vijaya Majumdar, Sanjib Patra, Akshay Anand

**Affiliations:** ^1^Division of Yoga and Life Sciences, Swami Vivekananda Yoga Anusandhana Samsthana (S-VYASA), Bengaluru, India; ^2^Neuroscience Research Lab, Department of Neurology, Post Graduate Institute of Medical Education & Research, Chandigarh, India; ^3^Department of Yoga, Central University of Rajasthan, Ajmer, India; ^4^CCRYN - Collaborative Centre for Mind Body Interventions Through Yoga, Post Graduate Institute of Medical Education & Research, Chandigarh, India; ^5^Centre of Phenomenology and Cognitive Sciences, Panjab University, Chandigarh, India

**Keywords:** cognitive impairment, yoga, prison, elderly, ageing crisis

An incorrect **Funding** statement was provided. The correct **Funding** statement reads: The author(s) declare financial support was received for the research and/or publication of this article. This work was funded by Swami Vivekananda Yoga Anusandhana Samsthana (S-VYASA), Bengaluru, India.

The original version of this article has been updated.

